# High‐Performance Bio‐Glue From the Berries of a Parasitic Plant

**DOI:** 10.1002/adma.74322

**Published:** 2026-07-29

**Authors:** Ufuk Gürer, Jana T. Reh, Jonas Röder, Umut Günsel, Chiara Gunnella, Lisa Schöbel, Franz Hagn, Aldo R. Boccaccini, Oliver Lieleg

**Affiliations:** ^1^ Department of Materials Engineering School of Engineering and Design Technical University of Munich Garching Germany; ^2^ Center For Functional Protein Assemblies (CPA) Munich Institute of Biomedical Engineering (MIBE) Technical University of Munich Garching Germany; ^3^ Institute of Biomaterials University of Erlangen‐Nuremberg Erlangen Germany; ^4^ Department of Bioscience Structural Membrane Biochemistry and Bavarian NMR Center (BNMRZ) School of Natural Sciences Technical University of Munich Garching Germany; ^5^ Molecular Targets and Therapeutics Center (MTTC) Institute of Structural Biology Helmholtz Munich Neuherberg Germany

**Keywords:** DES, mistletoe, polysaccharide, sticky, sustainable, tannic acid, wood adhesive

## Abstract

Most commercial adhesives are still petroleum‐based. Despite extensive efforts to develop sustainable alternatives, bio‐based adhesives often require chemical modification of their components, or they fail to achieve rapid, high‐performance bonding. Here, we report a fully bio‐based adhesive derived primarily from a (poly)saccharide extract obtained from the berries of the hemi‐parasitic plant mistletoe (*Viscum album*). This (poly)saccharide extract is supplemented with tannic and malic acid without any further chemical functionalization. Through a combination of heat‐treatment and rapid curing, the developed adhesive exhibits remarkable bonding strength across diverse substrates—including various types of wood and metal, glass, and polytetrafluoroethylene (Teflon)—and under various conditions. Furthermore, if samples are separated by force, the remaining adhesive layers can be reactivated via heat‐treatment to allow for efficient re‐bonding of the adherends. The fully bio‐based formulation not only enables multiple repair cycles but also valorizes a parasitic plant, thereby serving as an eco‐friendly, high‐performance alternative to petroleum‐derived adhesives.

## Graphical abstract

We report a fully bio‐based adhesive derived primarily from a (poly)saccharide extract obtained from the berries of the hemi‐parasitic plant mistletoe (*Viscum album*), supplemented with tannic and malic acid without any further chemical functionalization. Through a combination of heat‐treatment and rapid curing, the developed adhesive exhibits remarkable bonding strength across diverse substrates and under various conditions.

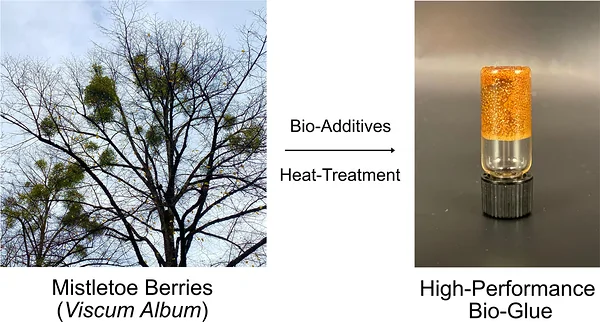

## 1 Introduction

Adhesives are used in almost every aspect of our daily life, from household activities to arts, crafts, and the industrial production of goods to large‐scale applications in skyscraper construction. The adhesive and sealant market is estimated to reach $90 billion by the end of the 2020s [1]. However, adhesives used today are mostly (about 80%) petroleum‐based products [2], and a majority of those either contain formaldehyde (e.g., urea‐formaldehyde) [3] or can later degrade into formaldehyde (e.g., cyanoacrylates) [4], which poses significant health hazards [5, 6]. Moreover, production and disposal of certain adhesives (e.g., of thermosets) can cause severe environmental damage since most adhesives form irreversible bonds; as a consequence, products made from different materials that are bonded using glues are difficult to recycle, which leads to the generation of more than 100 million tons of adhesive‐bonded waste each year [7, 8, 9].

To decrease and potentially eliminate our reliance on petroleum‐based sources, bio‐based materials from renewable feedstocks such as cellulose [7, 10], xylan [11], starch [12], and bio‐oil [13] have recently been explored as main components in sustainable adhesive formulations. However, whereas bio‐based systems can exhibit high adhesion strength, their synthesis often still relies on potentially hazardous reaction reagents, including redox salts (such as sodium periodate and sodium borohydride), epoxidation agents, and catalysts—all of which must be removed from the final product for safety reasons, thus rendering the overall production process time‐consuming and costly [11, 14]. In addition, organic solvents such as chloroform and N,N‐dimethylformamide are sometimes required during the fabrication process of the adhesive [15, 16]; the use and disposal of these chemicals raise concerns in terms of ecological impact and human health [11, 17]. Accordingly, the development of fully sustainable adhesives (including a sustainable production process) and fully recyclable final products is urgently needed [6, 9, 18]. Therefore, significant research is currently being conducted to develop purely bio‐based adhesives.

Many existing formulations—whether derived from sugar‐, polyphenol‐, or bio‐oil‐based sources—show promising adhesion properties [19, 20, 21, 22]; however, most of the bonding processes of the two adherends (typically, wood used for flooring and interior walls) still need to be completed with hot pressing at elevated temperatures up to 180°C to achieve high strength [3, 23, 24]. Among promising candidates for high‐strength adhesives, deep eutectic solvent (DES)‐based adhesives show great potential. For instance, a formulation based on classical DES components (urea and choline chloride) supplemented with poly(acrylic) acid can achieve a high lap shear strength up to ∼12 MPa—yet a toxic photoinitiator is required for curing [25]. As a greener alternative, a bio‐based DES adhesive combining cyclodextrin with acid components eliminates this toxicity issue by relying on network formation through hydrogen bonding [26]. However, in this particular formulation, the absence of covalent crosslinks limits the adhesion strengths to ∼6.6 MPa. An interesting recent study incorporated a lignin framework into the cyclodextrin‐based bio‐DES, utilizing ether bond linkages to form a robust chemical network. This lignin‐based adhesive showed an adhesion strength of ∼6.3 MPa on wood and was able to resist extreme conditions [27]. Nevertheless, the formulation requires hours of curing at temperatures up to 170°C, which is a similar bottleneck as found for bio‐oil (e.g., epoxidized soy oil)‐based adhesives, among these, a system recently reported by Westermann et al. [28] achieved remarkable adhesion on various substrates when the adhesive was cured at 180°C for 6 h.

**FIGURE 1:**
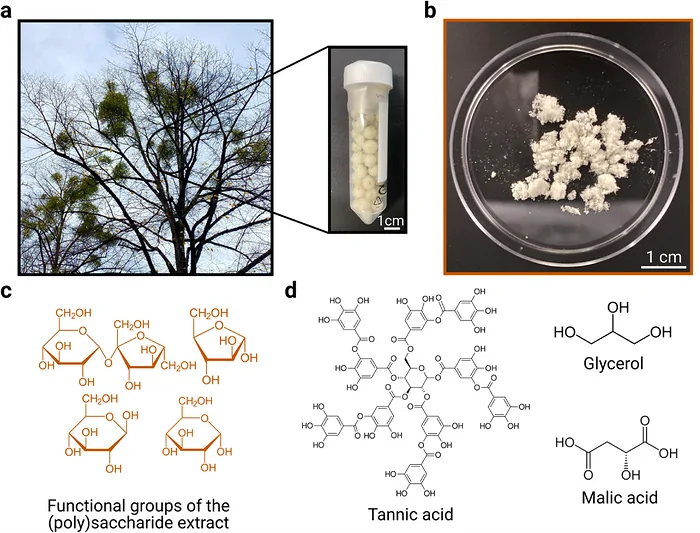
Components used to formulate a fully bio‐based, high‐strength adhesive. (a) Mistletoe plants growing on a tree and their berries (photo taken in Munich, Germany). (b) The freeze‐dried (poly)saccharide extract obtained from mistletoe berries and (c) its saccharide building blocks. (d) Supporting additives required to form the adhesive.

As an alternative, recently, mistletoe mucilage has gained attention as an inspiration for designing sustainable, bio‐based adhesive systems [29]. The European mistletoe, an evergreen parasitic plant, attaches itself to a broad range of host trees (Figure 1a); here, it grows uncontrollably while drawing water and nutrients from its host. This parasitism weakens the host tree, hinders its growth, flowering, and fruiting abilities, and can even lead to its death, thus posing a serious threat to forest ecosystems [30]. George et al. [29] investigated the composition of such mistletoe berries (Figure 1a) and identified highly sticky, water‐soluble saccharides attached to coiled cellulosic fibers. These structures provide mechanoresponsive adhesion that helps the seeds stick to host branches for germination. When a (poly)saccharide extract produced from such mistletoe berries is employed as an adhesive (without any further additives or heat‐treatment), it can achieve a considerable adhesive strength of ∼2.8 MPa on wood substrates; most likely, this is due to the functional groups of arabinofuranose and galactopyranose chains in combination with abundant monosaccharides. This adhesive strength is comparable to that of other sugar‐based glues reported in the literature, such as those made from glucose [23] or glucose oxidase [19].

In this study, we build on this previous work and introduce a fully bio‐based adhesive that reaches adhesive strengths of up to 10 MPa. Whereas adhesives mimicking the strategies employed by DES systems or carbohydrate‐acid networks typically rely on highly purified, single‐component sugars, we utilize an existing biological matrix. Specifically, we directly combine the crude (poly)saccharide extract from mistletoe berries (Figure 1b), which retains its natural, heterogeneous mixture of free monosaccharides (e.g., free glucopyranose), monosaccharide residues (e.g., arabinofuranose units; Figure 1c), and polyols, with tannic acid, malic acid, and glycerol (Figure 1d). Instead of synthesizing a formulation from isolated ingredients or chemically modifying one of the components prior to mixing, this approach repurposes the intact, unrefined botanical extract and turns it into a complex functional platform. Subsequent heat‐treatment of this mixture and curing during the gluing process induce the formation of a stiff supramolecular network, leading to strong lap shear bonding on various substrates without the need for hot pressing. Furthermore, the adhesive resists organic solvents quite well and can be reused after mechanical failure by reactivating it through a heat‐treatment, which further underlines the sustainability of the adhesive.

## 2 Results and Discussion

A sodium dodecyl sulfate‐polyacrylamide gel electrophoresis (SDS‐PAGE, stained with Coomassie Blue) conducted with a (poly)saccharide extract obtained from mistletoe berries (Figure S1) shows no detectable protein bands even though three lectin classes with a molecular weight around 30 kDa are typically observed in mistletoe berries [31]. This indicates that, after (poly)saccharide extraction, these lectins are either absent or present in trace amounts only. Consistent with this observation, a Bradford assay performed with the same (poly)saccharide extract (Figure S2) shows only a very low total protein content of ∼1.0% (w/w). Together, these findings demonstrate that the (poly)saccharide extract generated here contains only very small amounts of proteinaceous components, which is in agreement with reports from the literature [32].

**FIGURE 2:**
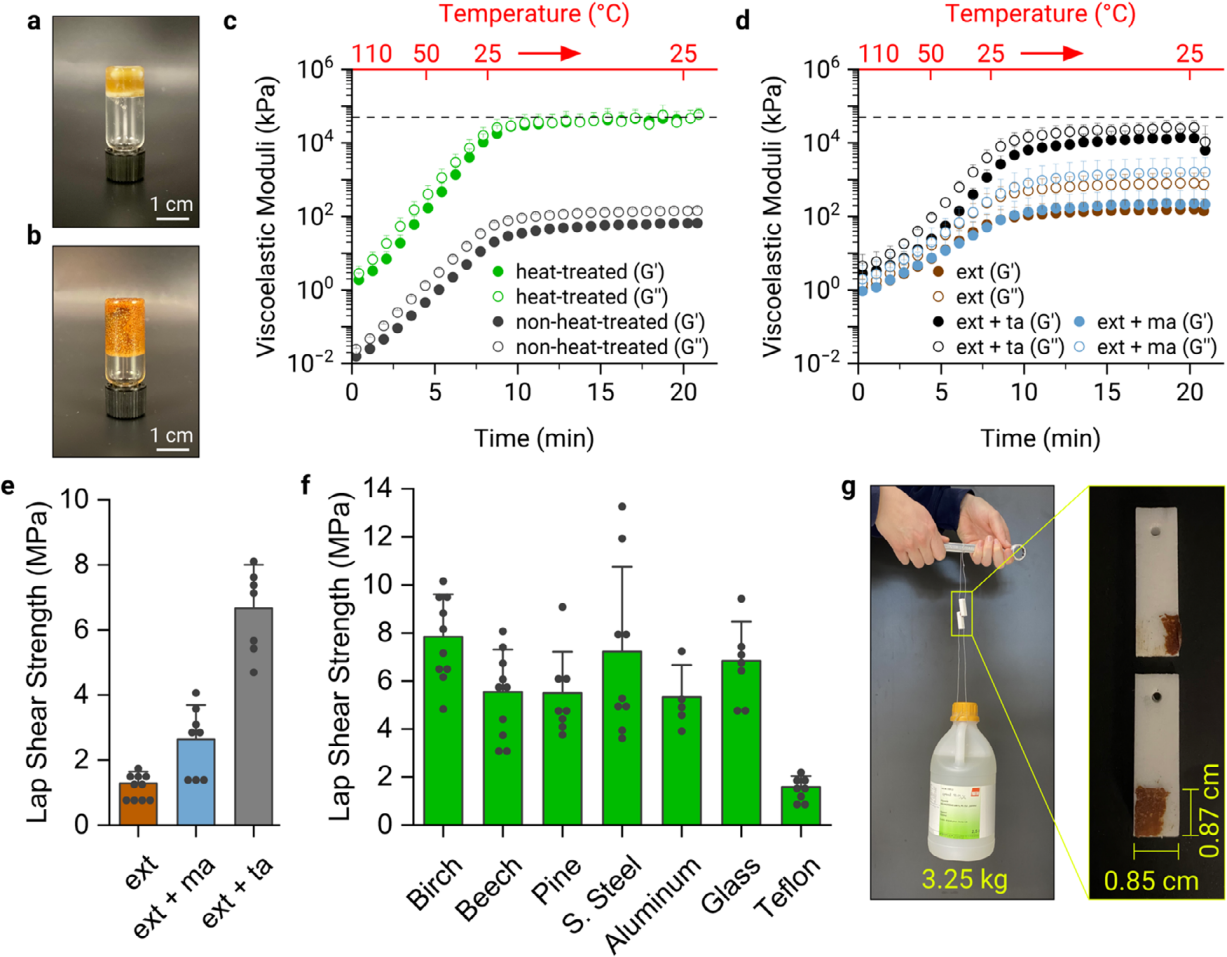
Characterization and performance of the adhesive and its components. Photos of the glue mixture (a) before and (b) after heat‐treatment. Viscoelastic properties of the full glue formulation (c) before (dark gray) and after heat‐treatment (green) and (d) of heat‐treated individual glue components: the mistletoe (poly)saccharide extract alone (brown), the extract in combination with malic acid (ma, blue), and the extract in combination with tannic acid (ta, black). In (c) and (d), the dashed line marks a level of 50 MPa. Data in (c,d) represents mean values; error bars denote the standard deviation as calculated from *n* = 3 samples. (e) Lap shear strength of the heat‐treated (poly)saccharide extract (brown), of the extract in combination with malic acid (blue), and of the extract in combination with tannic acid (gray) as determined on birchwood substrates. (f) Lap shear strength of the full glue formulation across various materials. Data in (e, f) represents mean values; error bars denote the standard deviation as calculated from *n* ≥ 5 samples. (g) Teflon specimens carrying a 3.25 kg bottle together with an image of the failed area of bonding (as triggered by a higher load).

Co‐dissolving this (poly)saccharide extract with tannic acid and malic acid produces a highly viscous, amber solution (Figure 2a). To form an adhesive by promoting supramolecular assembly and increasing intermolecular cohesion through the formation of a DES‐like structure, the mixture is tempered. However, even though tempering of sugar‐acid mixtures at 60°C has previously been reported to yield DESs [21], in our system, tempering at 60°C results in low‐strength adhesives. Therefore, we here select a higher temperature of 80°C for further processing, as temperatures above this point cause charring and render the material unusable. After tempering for 18 h at 80°C, the amber solution transforms into a darker and very stiff material (Figure 2b), indicating the successful solidification of the mixture.

Next, we analyze the alterations in the viscoelastic properties of the mixture during its fabrication process, that is, we mimic the heat‐treatment and subsequent rapid cooling to room temperature in situ while recording the viscoelastic moduli *G*′ and *G* *″*. As shown in Figure 2c, directly after heat‐treatment, the formulation exhibits a storage modulus (*G*′) of ∼1.7 kPa, which is about 100 times higher than that obtained for the same mixture without heat‐treatment (∼15.0 Pa).

At this point of the fabrication process, both material variants still exhibit the properties of a viscoelastic fluid with the loss modulus (*G″*) dominating the material response. During the cooling phase from 130°C to 25°C (which is conducted over a time interval of 8 min), both samples show strong increases in *G’* and *G″*, and both moduli approach a plateau shortly after the final temperature level of 25°C is reached. In this plateau region, the heat‐treated sample has a remarkably high storage modulus of ∼60 MPa, whereas the non‐heat‐treated sample only reaches ∼60 kPa. In other words, the 18 h heat‐treatment results in samples with 1000 times higher stiffness. With this realization in mind, for the remainder of this study, we always apply this heat‐treatment to all samples.

Importantly, control experiments show that both additives, malic acid and tannic acid, are required to achieve these high stiffness values. If either component is removed from the mixture, the final stiffness of the material is lower compared to when all reactants are present (Figure 2d), even after appropriate heat‐treatment: Combining the (poly)saccharide extract with tannic acid only results in *G’* values of ∼10 MPa, whereas the extract alone or combining it with malic acid only results in *G’* values of ∼200 kPa. Here, those simple mixtures have similar viscoelastic moduli right after heat‐treatment but lack the steep increase in stiffness during the cooling phase that is observed when all components are present.

Interestingly, when conducting lap shear tests using the different formulations to glue wood adherends together, we obtain similar trends as observed in rheology (Figure 2e,f). Both the (poly)saccharide extract alone (∼1.3 MPa) and its combination with malic acid (∼2.6 MPa) show relatively low adhesion strengths, whereas the extract mixed with tannic acid achieves a substantially higher adhesive strength (∼6.7 MPa). With the full mixture containing all three components, however, we obtain the highest adhesive strength (∼7.8 MPa). Moreover, formulations lacking tannic acid all display ductile fracture and creep‐like deformation (Figure S3), whereas samples containing tannic acid show a sharp, brittle fracture behavior. We also note that mixtures without tannic acid dry and solidify much more slowly, resulting in longer processing times until their full adhesion strength is reached. This behavior might be attributable to the hygroscopic nature of the extract [29] and malic acid [33]. Taken together, these observations suggest that tannic acid acts as the primary crosslinker in the formulation and critically contributes to the adhesive strength of the mixture.

**FIGURE 3:**
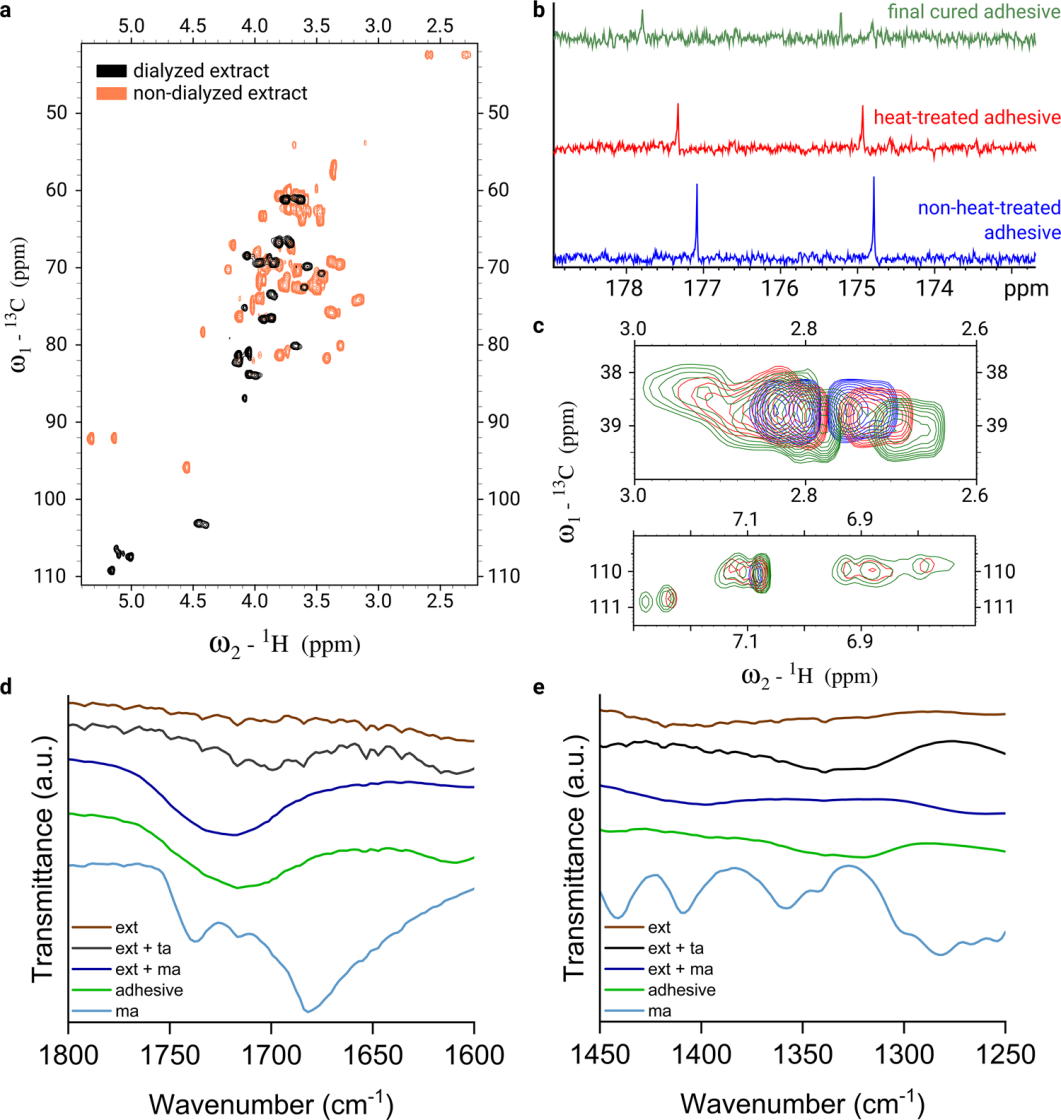
NMR and Fourier transform infrared spectroscopy (FTIR) analyses of the educts and the adhesive mixtures generated from them. (a) 2D‐[^13^C, ^1^H]‐HSQC spectrum of the (poly)saccharide extract before (coral) and after dialysis (black). The crude (poly)saccharide extract shows both monomeric/low‐molecular‐weight sugars (sharp, intense cross‐peaks) and polymeric units. After dialysis, the sharp signals from the smaller sugars disappear, and only the broad polysaccharide signals remain. (b) 1D‐^13^C spectrum of the full adhesive mixture without any heat‐treatment (blue), after 18 h of heat‐treatment at 80°C (red), and after curing the heat‐treated sample at 130°C for 20 min (green). (c) Selected regions of the 2D‐[^13^C, ^1^H]‐HSQC spectrum of the full adhesive, focusing on the malic acid region (upper panel) and the tannic acid region (lower panel). The color scheme used in (c) matches the one used in (b). The full HSQC spectrum of this measurement is provided in Figure S6. Zoomed‐in views of the (d) carbonyl region and the (e) C─O/O─H region of the FTIR spectra obtained for the full adhesive and a subset of its components. The raw spectra are unsmoothed and, for better comparison, normalized to a range between 0 and 100.

To obtain a better understanding of the (poly)saccharide composition and the chemical behavior of the adhesive mixture after heat‐treatment and curing, a set of nuclear magnetic resonance (NMR) spectroscopy analyses is conducted. The 2D‐[^13^C, ^1^H]‐HSQC spectrum of the (poly)saccharide extract (Figure 3a) is dominated by correlations originating from monosaccharides. Here, the most prominent signals arise from α‐ and β‐D‐glucopyranose, with anomeric cross‐peaks observed at δH/δC 5.17/92 ppm and 4.70/95 ppm, respectively [34]. Additional signals attributed to ring protons and carbons of these two variants of glucopyranose are located within the characteristic carbohydrate region (δH 3.1–3.9 ppm and δC 58–78 ppm) [34, 35]. This region contains further resonances, possibly indicating contributions from polyols (e.g., pinitol, ononitol), which are characteristic components of mistletoe berry extracts [29]. In addition, we observe small NMR signals at 5.15 H/109 C, 5.1 H/107 C, and 5.0 H/107 C (Figure 3a; Figure S4), which most likely correspond to arabinofuranose groups [29, 36]. To further characterize the (poly)saccharide structure in the extract, it is dialyzed, after which additional peaks become visible in the anomeric carbon region (Figure 3a), indicating multiple glycosidic linkages. The signals at ∼4.4 H/105 C correspond to β‐galactopyranose, whereas the peaks at 5.15 H/109 C, 5.1 H/106 C, and 5.0 H/107 C—assigned to terminal and linked α‐arabinofuranose units—intensify relative to the non‐dialyzed (poly)saccharide extract [29]. These new observations, together with the altered signals in the carbohydrate region of the dialyzed (poly)saccharide extract, align very well with previously proposed polysaccharidic signatures (i.e., arabinogalactans) of mistletoe berry extracts [29, 32].

Furthermore, we detect a set of resonances at δH 3.9–4.1 ppm and δC 74–82 ppm, as well as at δH 5.35/δC 92 (Figure 3a; Figures S5 and S6). These signals may originate from a furanose‐type sugar (i.e., fructose) present in the form of sucrose [35, 37], which remains stable when the berry extract is heat‐treated in the absence of any additives (Figure S5). However, the same resonances become thermally unstable and disappear under identical heat‐treatment when malic acid and tannic acid are present (Figure S6). This transformation is accompanied by the appearance of a new ^1^H signal at ∼9.3 ppm (Figure S7), which might be attributable to the formation of trace amounts of aldehyde‐containing units, that is, degradation products of fructose [38].

Having characterized the extract, we next perform further NMR analysis of the adhesive mixture together with its heat‐treated variant and the final, cured form (Figure 3b,c). In the ^13^C spectrum, the two carboxyl carbons of malic acid shift downfield from ∼177.1 and ∼174.8 ppm to ∼177.7 and ∼175.2 ppm, respectively (which is accompanied by a decreased peak intensity). Interestingly, an additional, weak signal appears near ∼174.9 ppm (Figure 3b). Similarly, the signal for the CH_2_ moiety in malic acid, which is originally observed between δH 2.74 and δH 2.83 ppm (Figure 3c), is progressively shifted upfield after the heat‐treatment and the curing step. A slight downfield shift is observed for the corresponding carbon atom, whose signal shifts from ∼38.6 to ∼39.1 ppm (Figure 3c), with a small residual peak forming near δH 2.9/δC 38.6 ppm. Moreover, the enlarged galloyl region of tannic acid in the 2D‐[^13^C, ^1^H]‐HSQC spectrum (Figure 3c) reveals new signals after the heat‐treatment and the curing step, which indicates a degradation of tannic acid.

Evidence for such a thermal degradation of tannic acid is also observed in data obtained from mass spectrometry. The mass spectrum of thermally untreated tannic acid is characterized by a variety of peaks, notably at 483, 635, 787, 938, and 1090 m/z, corresponding to di‐, tri‐, tetra‐, penta‐, and hexagalloyl glucose, respectively (Figure S8). Among these, pentagalloyl glucose exhibits the highest signal intensity. In the lower mass region, prominent peaks observed at 169 and 321 m/z (Figure S9) are attributable to gallic acid and digallic acid fragments [39]. Upon dissolution of tannic acid within the mistletoe extract (with water and glycerol but without the addition of malic acid) followed by heat‐treatment, distinct shifts in peak intensities occur. Specifically, the relative abundance of tetragalloyl glucose (787 m/z) increases compared to pentagalloyl glucose (938 m/z), further supporting the partial thermal degradation of tannic acid (Figure S10). Upon heat‐treatment, the ester bond in the pentagalloyl glucose structure connecting the galloyl unit to the anomeric carbon of glucose is more likely to be attacked by alcohols (such as glycerol) or water molecules. For this reason, this structure is quite unstable, and its abundance is reduced compared to that of tetragalloyl‐glucose (whose relative abundance remains similar). Interestingly, tannic acid does not completely hydrolyze into gallic acid monomers; it retains larger oligomeric structures, which potentially contribute to the mechanical stability and viscosity of the adhesive.

In addition to these alterations in peaks already present for pure tannic acid, new signals emerge in the tannic acid/mistletoe extract sample at 243 and 486 m/z (Figure S11), which may point toward an ester formation between glycerol and gallic acid [40]. Furthermore, those newly formed peaks (e.g., 243 m/z) are also present when the full adhesive mixture is investigated after heat‐treatment (Figure S12) even though the spectrum is now dominated by the malic acid signal at 133 m/z. Here, the signal at 295 m/z might represent the reaction product between malic acid and glucose, while the signal at 207 m/z could be associated with an ester formation between malic acid and glycerol [41].

This notion of ester formation is further supported by an analysis of the obtained FTIR spectra (Figure 3d,e). Our results indicate that malic acid is consumed under the reaction conditions employed here. Specifically, after heat‐treatment, the double peaks corresponding to the carbonyl (C═O) stretching of the –COOH group of malic acid (Figure 3d) at approximately 1735 and 1680 cm^−1^ are not present anymore in both the full adhesive formulation (green curve) and in the mixture of mistletoe extract and malic acid. Simultaneously, a new broad band emerges at around 1720 cm^−1^ in the carbonyl region (Figure 3d), indicating the possible formation of new ester groups. This finding is further supported by the reduction of the (O─H) bending and (C─O) stretching bands of malic acid carboxylic acid groups (Figure 3e) located at approximately 1410 and 1280 cm^−1^, respectively, which supports our esterification hypothesis [23, 28]. Based on these findings, it is reasonable to assume that the resulting supramolecular network is highly crosslinked via covalent interactions and hydrogen bonds.

Next, we conduct additional lap shear tests with the complete adhesive formulation by testing it on other substrates (Figure 2f) and under various conditions (Figures S13–S15). On beech and pine wood samples, we obtain similar adhesive strengths of ∼5.5 MPa, which are lower than the result obtained on birch wood (∼7.8 MPa). Yet, in all three cases, the adhesive predominantly exhibits brittle fracture (Figure S16). For these wood variants, the observed high lap shear strengths are likely due to the penetration of the adhesive into the porous wood structure (Figure S17), which results in a stronger interfacial bond. On stainless steel, the adhesive yields an average lap shear strength of ∼7.2 MPa (Figure 2f). Given its frequent exposure to diverse environmental conditions in industrial applications, we also evaluate the effect of surface contamination by coating the steel surfaces with carbon black particles prior to bonding (Figure S13). Despite this severe contamination, the average lap shear strength remains remarkably high at ∼6.8 MPa. Similarly, we observe robust adhesion on aluminum samples (∼5.3 MPa), which remains at a decent level even on rough aluminum samples (∼4.0 MPa, Figure S14). Moreover, we test glass adherends, which should offer a high potential for forming hydrogen bonds between surface oxides and the adhesive, and we obtain a high lap shear strength of ∼6.8 MPa here (Figure 2f). Importantly, this value stays at a similar level when the adhesive is applied to wet glass surfaces (∼7.3 MPa, Figure S15), and bonded glass samples even remain stable after exposure to cryogenic temperatures (where we find an average lap shear strength of ∼3.7 MPa; Figure S15). Lastly, on Teflon samples, we find an average adhesion strength of ∼1.6 MPa (Figure 2f), which is the lowest value among the different materials tested here but still sufficient to carry a load of more than 3.0 kg (Supporting Video) with a bonded area of less than 0.75 cm^2^ (Figure 2g; here, the bonded area was determined after forcing the Teflon samples to fail by applying a larger load). A visual inspection of the failed specimen reveals an adhesive failure for this material (Figure 2g), suggesting that the cohesive forces within the adhesive might dominate over the interfacial adhesion to Teflon.

**FIGURE 4:**
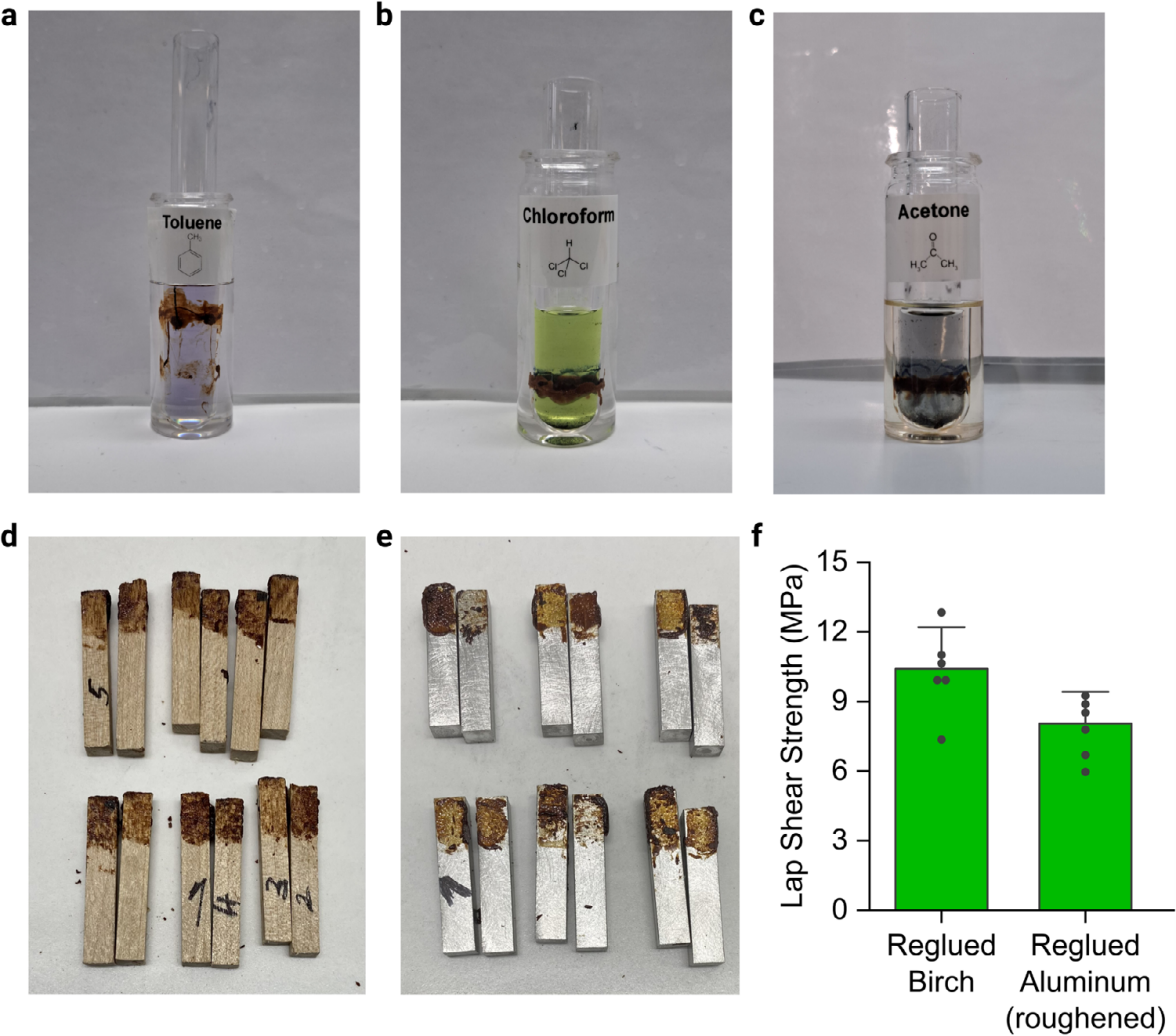
Stability assessment and reusability of the adhesive. (a–c) Broken glass tubes, where the glass interface was mended using the adhesive, filled with a (colored) organic solvent and placed into a beaker containing the same solvent: (a) toluene (colored with diluted Sudan black B, C.I. 26150); (b) chloroform (colored with a mixture of Coomassie Brilliant Blue G‐250, Applichem, Germany, and yellow food coloring, RUF, Quakenbrück, Germany); (c) acetone (colored with diluted Coomassie Brilliant Blue G‐250). Photos were taken after an incubation time of 1 week. (d–e) Photographs of glued samples after failure was enforced during lap shear tests: glue residues are clearly visible on both sides of (d) birch wood and (e) roughened aluminum samples. (f) Lap shear strength values of repaired/reglued birch wood and aluminum samples. For these tests, 12 individual adherends selected from six failed sample pairs were randomly paired for re‐bonding. Bars represent mean values; error bars denote the standard deviation as calculated from *n* = 6 samples.

Given that the adhesive itself is highly polar, we hypothesize that it can resist non‐polar organic solvents. To test this notion, we mechanically sever the tips of glass tubes, glue those broken pieces back together at the fracture site, and fill them with common organic solvents; in detail, we test three solvents, and those are—listed in increasing order of polarity [42]—as follows: toluene, chloroform, and acetone. After 1 week of incubation, we observe that the adhesive demonstrates excellent resistance and stability toward toluene and chloroform (Figure 4a,b). In contrast, although the bonded glass interface remained intact, minor glue degradation occurred after incubation in acetone (Figure 4c), and this result is likely due to the higher polarity of acetone compared to the other two solvents.

Since most of the components used to produce the adhesive have been reported to possess antimicrobial activity [43, 44, 45], we test whether the adhesive formulation exhibits such antibacterial effects. For those tests, we select *B. subtilis* as a model strain for Gram‐positive bacteria, and *E. coli* as a model strain for Gram‐negative bacteria. When comparing the inhibition zones after incubating the bacteria on agar plates at 80% humidity for 1 month, it is observed that *B. subtilis* is inhibited from growing in the direct vicinity of the glue (Figure S18). Similarly, *E. coli* does not grow on top of the glue layer; however, colonies are detected in close proximity to the adhesive. This indicates that the adhesive might potentially exert a stronger effect toward *B. subtilis* than for *E. coli*. Nevertheless, owing to the partial dissolution and diffusion of the adhesive into the agar plate, driven by both the high humidity of the incubator and the long duration of incubation, a quantification of the occurring inhibition zones is challenging. Therefore, we conduct endpoint tests with bacteria solutions that were challenged with the adhesive. Representative photographs of the agar plates depict a visibly higher colony‐forming unit (CFU) count for *B. subtilis* incubated in the medium alone compared to samples exposed to the adhesive (Figure S19). This visual difference is accompanied by a reduction in CFUs by one order of magnitude (Figure S19), matching our findings on inhibition zone formation. Together, these results demonstrate an antibacterial behavior of the adhesive toward *B. subtilis*. However, for the same dilution assessed in this experiment, there is no observable reduction in CFUs for *E. coli* when incubated together with the adhesive compared to its respective control (Figure S19).

Moreover, we hypothesize that the adhesive can be reused after mechanical failure, as we observe glue residues on both sides of the samples (Figure 4d,e). To do so, we place the wood adherends at 100°C for 10 min to ensure sufficient liquefaction of the glue residues. Afterward, we re‐bond them and test their lap shear strength. Here, almost all reglued wood samples exhibit similar or even higher lap shear strength values compared to what we obtained during the initial round of mechanical tests (Figures 2f and 4f), sometimes exceeding 10 MPa. This behavior may be attributable to the additional heating step, which potentially improves the crosslinking density within the adhesive. After these tests, we reglue the failed wood samples once more, employing the exact same conditions as during the first regluing cycle; now, the resulting lap shear strengths are lower but still remain above 6 MPa (Figure S20). Similarly, also re‐bonded roughened aluminum samples perform well in the lap shear tests; for these samples, reheating the glue residues at a lower temperature of 90°C for 10 min is sufficient. Finally, to evaluate the feasibility of other heating techniques beyond conventional oven setups, we also explore local heat generation with a heat gun. Interestingly, when working with heat‐conductive adherends, even a very short (40 s) exposure to localized heat results in good lap shear strengths of ∼1.8 MPa (Figure S21). This demonstrates that the adhesive can be successfully cured via a rapid, out‐of‐oven approach, thereby significantly broadening its versatility and applicability in real‐world settings.

## 3 Conclusion

In summary, a high‐strength adhesive is successfully produced without chemically modifying any of the components or using toxic chemicals. Instead, sustainable additives enhance the highly sticky (poly)saccharide extract obtained from the berries of mistletoe plants, and molecular interactions between the different components are triggered by a combination of a heat‐treatment and curing. For this adhesive to be used in an application, only the curing is necessary, and bonding two adherends by hand is sufficient to obtain the high adhesive strengths described here. This highly time‐efficient process, making use of a material fully made from biological components, gives rise to remarkable adhesion properties across various surfaces and conditions. Future research should investigate the full antimicrobial potential of the adhesive, its long‐term stability, and different curing options of this bio‐adhesive to fully elucidate its potential to replace conventional, petroleum‐based glues.

## 4 Experimental Section

If not stated otherwise, all chemicals were purchased from Carl Roth GmbH (Karlsruhe, Germany).

### 4.1 (Poly)Saccharide Extraction

Water‐soluble (poly)saccharides attached to the coiled cellulose fibrils within the berries of European mistletoe were extracted as described in the following. First, the fibrils were carefully separated from the berry flesh with tweezers by pulling out the seed and opening the fibrillar structure. The separated fibrils were then thoroughly washed with distilled water. To further increase the concentration of soluble (poly)saccharides, the remaining berry flesh was also immersed in the same water. After incubating approximately 30 berries in 2 L of distilled water, the heterogeneous mixture was stirred overnight to maximize the solvation of (poly)saccharides. The mixture was then centrifuged (Centrifuge 5430, Eppendorf) at 7830 rpm for 20 min. The supernatant was collected and filtered through cellulose filter paper with a pore size of 2−3 µm, yielding a transparent solution. This solution was freeze‐dried and stored at −70°C until further use. To increase the concentration of the polysaccharides in this solution by removing smaller molecules from the extract, a batch of the extract was dialyzed against distilled water for 2 days at RT using a porous membrane (MWCO = 12−14 kDa, Spectra/Por). The dialyzed extract was then freeze‐dried and stored at ‐70°C until further use.

### 4.2 Preparation of the Adhesive

The lyophilized mistletoe (poly)saccharide extract (230 mg), tannic acid (80 mg, MW = 1701.20 g mol^−1^, puriss., powder, Sigma‐Aldrich), and malic acid (80 mg, MW = 134.09 g mol^−1^, DL, purity ≥ 99%) were dissolved in 75 µL of deionized water and 50 µL of glycerol (≥ 99.5%, Sigma‐Aldrich) at 80°C and 100 rpm. The selection of malic acid (which could be exchanged with citric acid) and the mass ratio of malic acid to tannic acid were determined based on pre‐tests (Figure S22) and the solubility limits of the components within the matrix. During the dissolution process, the mixture was centrifuged (at 4000 rpm for 2 min) at several time points to test for complete dissolution of all ingredients. After full dissolution, the resulting viscous solution was placed in an oven at 80°C for 18 h without a lid. As control samples, the following formulations were used: the extract alone (230 mg), 230 mg extract + 80 mg tannic acid, 230 mg extract + 80 mg malic acid; those mixtures were prepared following the same procedure as described above.

### 4.3 Application of the Adhesive

After the 18 h heat‐treatment (see above), the adhesive was smeared onto the surfaces of two birch wood adherends (5 × 5 × 30 mm^3^). These adherends were then placed into an oven set to 130°C. After 20 min of incubation, a darker adhesive layer was observed on the treated surfaces. The adherends were subsequently bonded and allowed to cool down for 50 s, after which the bonded assembly was manually pressed for 10 s. The same procedure was applied to beech and pine wood (6 × 6 × 30 mm^3^), stainless steel (1.4301, 6 × 6 × 30 mm^3^), smooth and roughened aluminum alloy (AlCuMgPb F37, 6 × 6 × 30 mm^3^; the roughened version was prepared by treatment with 120‐grit sandpaper), glass (4 × 5 × 25 mm^3^), and Teflon (which was treated with 60‐grit sandpaper, 9 × 9 × 30 mm^3^). However, to bond metal and Teflon adherends, the parts were pressed together for about 1 min after the adhesive cooled down. Then, the specimens were subjected to compression tests. Wood samples were dried in an oven prior to bonding. Glued samples were placed in silica beads to enhance the drying process. With the exception of the cryogenic tests, all samples were tested under dry conditions.

### 4.4 Mechanical Performance

Lap shear specimens were tested under compression using a universal testing machine (Instron 5967, Instron, Germany) equipped with a 30 kN load cell. Almost all specimens were mounted into the Instron device via a custom‐made sample holder (Figure S23) with their long axis parallel to the loading direction; then, they were compressed at a speed of 1.0 mm min^−1^ until failure, while the resulting force‒displacement curves were recorded. Only for the highly brittle glass samples, stabilization was conducted without the sample holder to avoid the formation of cracks in the adherend. Here, a metal plate with a slit was placed onto the sample plate of the device into which one end of the glued sample was inserted—and this prevented sliding during compression testing. To calculate the lap shear strength from the recorded data, the dimensions of the bonded area were measured with a caliper before conducting the lap shear tests.

### 4.5 Bradford Assay

The aqueous (poly)saccharide extract solution (1.0 mg mL^−1^) was mixed in a well plate with Bradford reagent (Thermo Fischer Scientific) in a 1:1 (v/v) ratio. After 5 min of incubation, sample absorbance at 595 nm was measured using a microplate reader (Varioskan LUX, ThermoFisher Scientific, Vantaa, Finland). The same procedure was applied to aqueous solutions of bovine serum albumin (Fraction V) solutions of concentrations of 0.005, 0.01, and 0.02 mg mL^−1^.

### 4.6 SDS‐PAGE

Gel electrophoresis was performed as described previously [46]. Briefly, the (poly)saccharide extract solution (2.0 mg mL^−1^) was mixed 1:1 (v/v) with 2X sample buffer (120 mM tris(hydroxymethyl)aminoethane hydrochloride (TRIS‐HCl), 4.0% (w/v) of SDS, 20% (v/v) glycerol, 0.02% (w/v) bromophenol blue sodium salt, pH = 6.8) and heated to 95°C for 5 min. Subsequently, 20 and 10 µL aliquots of the mixture were loaded onto a 10% precast polyacrylamide gel (Sigma‐Aldrich). The gels were run at 200 V for 30 min, with a protein marker (Precision Plus Protein Kaleidoscope Standards, Bio‐Rad) loaded into the first lane; protein bands were visualized with Coomassie Brilliant Blue solution.

### 4.7 Rheological Measurements

The viscoelastic moduli of mixtures were measured on a commercial shear rheometer (MCR 302, Anton Paar GmbH, Graz, Austria) equipped with an 8 mm (PP 08, Anton Paar) measuring head and a disposable tin‐coated bottom steel plate for single use. The samples were placed onto the bottom plate of the rheometer, which was then heated to 130°C. This heat level was maintained for 20 min to mimic the conditions during the gluing process. Afterward, the bottom plate was cooled to 25°C and kept at this temperature until the end of the measurement. Subsequently, the measuring head was lowered until the device registered a minimum normal force of 2.0 N to ensure full contact with the glue. Then, the viscoelastic material properties were recorded over time by applying torques around 70 µNm at a frequency of 1.0 Hz.

### 4.8 NMR Spectroscopy

Analyses were conducted on a Bruker Avance III spectrometer operating at a proton frequency of 500 MHz with a cryogenic probe. The experiments were controlled using Topspin 3.7 (Bruker, Germany), and data processing was performed using Topspin 4.1. Samples were dissolved at a concentration of 10.0 mg mL^−1^ in D_2_O (99.8%). The following experiments were recorded at 298 K: 1D‐^1^H, 1D‐^13^C, and 2D‐^1^H,^13^C‐HSQC, using ZGGPW5, ZGPG30, and HSQCETGPSP pulse programs, respectively, as provided in the Bruker pulse program library.

### 4.9 ATR (Attenuated Total Reflection)‐FTIR Spectroscopy

The adhesive as well as its components were analyzed using a Shimadzu IRAffinity‐1S (Shimadzu Corp., Japan) equipped with a Quest ATR GS10801‐B single bounce diamond accessory (Specac Ltd., England). Data were acquired, carrying out 40 scans at a resolution of 4 cm^−1^ applying Happ‐Genzel apodization; the obtained data were processed using the LabSolution IR software by Shimadzu.

## Author Contributions

**Ufuk Gürer**: conceptualization, investigation, writing – original draft, writing − review and editing, data curation. **Jana T. Reh**: investigation, writing – review and editing. **Jonas Röder**: investigation, writing − review and editing. **Umut Günsel**: investigation, writing − review and editing. **Chiara Gunnella**: investigation, writing − review and editing. **Lisa Schöbel**: investigation, writing – review and editing. **Franz Hagn**: supervision, resources, writing − review and editing, conceptualization. **Aldo R. Boccaccini**: conceptualization, resources, writing – review and editing, supervision. **Oliver Lieleg**: conceptualization, resources, writing − original draft, writing − review and editing, supervision, funding acquisition.

## Conflicts of Interest

The authors declare no conflicts of interest.

## Supporting information




**Supporting File 1**: adma74322‐sup‐0001‐SuppMat.docx.

**Supporting File 2**: adma74322‐sup‐0002‐supportingvideo.mov.

## Data Availability

Data supporting the findings of this study are provided in the supporting file. Additional data that support the findings of this study are available from the corresponding author upon reasonable request.
