# Supplementary material for: High‐Performance Bio‐Glue From the Berries of a Parasitic Plant
Source: Adv Mater. 2026 Jul 29;38(53):e74322. doi: 10.1002/adma.74322 (PMC13591666; doi:10.1002/adma.74322)
Supplement: Supplementary file 1 [file ADMA-38-e74322-s001.docx]

**Supporting Information for**

**High-Performance Bio-Glue from the Berries of a Parasitic Plant**

Ufuk Gürer^1,2^, Jana T. Reh^1,2,*^, Jonas Röder^3,*^, Umut Günsel^4,5^, Chiara Gunnella^1,2^,
Lisa Schöbel^3^, Franz Hagn^4,5^, Aldo R. Boccaccini^3^, Oliver Lieleg^1,2,#^

^1^ Department of Materials Engineering, School of Engineering and Design,
Technical University of Munich, Boltzmannstraße 15, 85748 Garching, Germany

^2^ Center for Functional Protein Assemblies (CPA), Munich Institute of Biomedical Engineering (MIBE), Technical University of Munich, Ernst-Otto-Fischer Straße 8, 85748, Garching, Germany

^3^Institute of Biomaterials, University of Erlangen-Nuremberg,
Cauerstraße 6, 91058 Erlangen, Germany

^4^Structural Membrane Biochemistry and Bavarian NMR Center (BNMRZ),
Department of Bioscience, School of Natural Sciences, Technical University of Munich,
Ernst-Otto-Fischer Straße 2, 85748, Garching, Germany

^5^Molecular Targets and Therapeutics Center (MTTC), Institute of Structural Biology, Helmholtz Munich, Ingolstädter Landstraße 1, 85764 Neuherberg, Germany

***:** equal contribution **#:** corresponding author

Prof. Dr. Oliver Lieleg

Center for Functional Protein Assemblies (CPA),

Technical University of Munich,

Ernst-Otto-Fischer Straße 8, 85748 Garching, Germany

E-Mail: oliver.lieleg@tum.de, Phone: +49 89 289 10952

*keywords: DES, wood adhesive, sustainable, mistletoe, polysaccharide, sticky, tannic acid*

*ORCID IDs*: **UfG** (0009-0004-0016-7406); **JTR** (0009-0005-1462-8311); **JR** (0009-0003-1920-2155); **UmG** (0000-0002-3109-4826); **CG** (0009-0008-1135-963X); **LS** (0000-0003-0391-4089); **FH** (0000-0002-1315-459X); **ARB** (0000-0002-7377-2955); **OL** (0000-0002-6874-7456)

**Figure S1.** SDS-PAGE of the (poly)saccharide extract showing the absence of proteins.

**Bradford assay:** The absorbance of the (poly)saccharide extract (0.5 mg mL^–1^, dissolved in distilled water) was determined to be 0.14 ± 0.0034. According to the albumin standard curve shown in Figure S2, this corresponds to a protein concentration of 0.0056 mg mL^–1^. Therefore, a 0.5 mg extract contains roughly 0.0056 mg of protein, which corresponds to ~1.0 % (w/w).

**Figure S2.** Albumin standard curve; the data shown represents average values as calculated from five measurements each.

**Figure S3.** Exemplary lap shear strength–displacement curves obtained on birch wood samples are shown for different adhesive formulations: ext (the (poly)saccharide extract alone), ext + ma (extract + malic acid), and ext + ta (extract + tannic acid). All mixtures were heat-treated and cured as described in the main text. The curves shown represent one representative measurement for each group.

**Figure S4**. 2D-[^13^C,^1^H]-HSQC spectrum of the (poly)saccharide extract before (coral) and after dialysis (black).

**Figure S5.** 2D-[^13^C,^1^H]-HSQC spectrum of the (poly)saccharide extract before (blue) and after 18 hours of heat-treatment at 80 °C (red).

**Figure S6.** 2D-[^13^C,^1^H]-HSQC spectrum of the freshly prepared adhesive mixture (blue), after 18 hours of heat-treatment at 80 °C (red), as well as after subsequently curing the heat-treated sample at 130 °C for 20 min (green).

**Figure S7.** ^1^H spectrum of the mistletoe (poly)saccharide extract and the full adhesive mixture under heat-treatments. Upon heat-treatment in the presence of malic acid and tannic acid, a new peak emerges at 9.3 ppm.

**Electrospray Ionization Mass Spectrometry (ESI-MS):** All samples were dissolved in pure water at a concentration of 0.1 mg mL^–1^. Then, mass spectrometric analysis of the products was performed in negative ion mode using an LCQ-Fleet system (Thermo Fisher Scientific) equipped with a 3D ion trap and an ESI source. The mass spectrometer was interfaced with an UltiMate 3000 High-Performance Liquid Chromatography (HPLC) system utilizing a loop injection method.

**Figure S8.** Mass spectrometric analysis of a tannic acid solution in the m/z range of 300 to 2000.

**Figure S9.** Mass spectrometric analysis of a tannic acid solution in the m/z range of 50 to 600.

**Figure S10.** Mass spectrometric analysis of the adhesive mixture without malic acid in the m/z range from 200 to 3000. The sample was analyzed after heat-treatment.

**Figure S11.** Mass spectrometric analysis of the adhesive mixture without malic acid in the m/z range from 50 to 600. The sample was analyzed after heat-treatment.

**Figure S12.** Mass spectrometric analysis of the full adhesive mixture in the m/z range from 50 to 600. The sample was analyzed after heat-treatment.

**Figure S13.** *Left:* Stainless steel samples covered with carbon black particles (carbon content >99.9% (w/w), Werth-Metall, Erfurt, Germany) to mimic dust contaminations of the surface. *Right:* Lap shear strengths obtained for such contaminated steel samples. The bar represents the mean value, the error bar denotes the standard deviation as calculated from *n* = 8 samples.

**Figure S14.** Adhesive performance on aluminum adherends, which were randomly roughened with 120-grit sandpaper. The bar represents the mean value, the error bar denotes the standard deviation as calculated from *n* = 7 samples.

**Figure S15.** Lap shear strength of bonded glass samples tested under two different conditions. The 'wet' condition represents results obtained from glass adherends immersed in water prior to bonding. The 'frozen' condition represents glued glass samples that were sequentially stored (for 24 h at each temperature) at -20 °C, -80 °C, and -150 °C prior to testing. Bars represent mean values, error bars denote the standard deviation as calculated from *n* ≥ 6 samples.

**Figure S16.** Exemplary lap shear strength-displacement curves obtained for the adhesive formulation are depicted for different wood types. The curves shown represent one representative measurement for each group.

**Light Microscopy:** To expose the glue layer between two bonded wood pieces so it is accessible for imaging, bonded wood samples were cut perpendicular to the glued interface using a microtome (SM2000 R, Leica, Nussloch, Germany). The interfacial region was then imaged on a reflected light microscope (Axioplan, Zeiss, Germany) using bright-field configuration and a 10X lens.

**Figure S17.** Optical microscopy analysis of the adhesive-wood interface at 10X magnification. Microscope images show birch, beech, and pine wood samples (a–c) after gluing, and (d–f) after 7 days of incubation at room temperature. The dark adhesive penetrates the porous wood structures, and this effect is more pronounced in the birch and pine variants. This phenomenon persists when the cut wood samples were imaged again after 7 days of incubation at room temperature. Orange rectangles indicate selected areas for comparison. Scale bars represent 200 µm.

**Bacterial inhibition zone test:** Agar plates containing 2.5% (w/v) Luria/Miller (LB) broth and 1.5% (w/v) agar-agar (Kobe I) were prepared and stored at 7 °C until use*.* *B. subtilis* and *E. coli* were incubated overnight in LB medium and subsequently diluted to an optical density at 600 nm (OD_600_) of ~0.085 and ~0.097, respectively. For each bacterial strain, a 25 µL aliquot of the diluted bacterial suspension was spread evenly across the surface of the agar layer. On the same day, adhesive samples (previously prepared on glass adherends) were separated from the adherend, trimmed, and placed into the center of the Petri dishes; control plates were maintained without adding adhesive samples. All plates were then incubated at 37 °C and 80% humidity for 1 month. Following incubation, photographic images of each petri dish were captured.

**Figure S18.** Inhibition zones on agar plates. Petri dishes were inoculated with (a – c) *B. subtilis* and (d – f) *E. coli* and cultivated in the presence of adhesive samples placed into the center of the petri dish. (a) and (d) show the control groups, where no adhesive was added. Scale bars represent ~1 mm.

**End-point tests with bacteria incubated in liquid culture:** To investigate the (putative) antibacterial activity of the adhesive, adhesive samples were prepared on glass substrates, trimmed, and weighed (ranging between 9.30 mg and 13.65 mg). Planktonic cultures of *B. subtilis* (as a model Gram-positive strain) and *E. coli* (as a model Gram-negative strain) were grown overnight in LB medium and subsequently diluted to an OD_600_ of ~0.071 and ~0.078, respectively. Each adhesive sample was then immersed in the bacterial suspension at a final adhesive concentration of 6 mg mL^‒1^ and incubated at 37 °C under gentle agitation. After 10 h of incubation, the bacterial suspensions were serially diluted (to a final dilution factor of 10^‒5^) and homogenized *via* vortexing. A 30 µL aliquot of each dilution was spread evenly across nutrient-enriched agar plates, which were then incubated at 37 °C and 80% humidity for 13 h. Following incubation, pictures were taken, and the software ImageJ (version 1.54g) was used to count the colony-forming units (CFUs) for both, samples incubated with the adhesive and control samples incubated in medium alone.

**Figure S19.** Bacterial growth end-point assay. Representative photographs showing CFUs of *B. subtilis* incubated (a) without and (b) with the adhesive, and of *E. coli* incubated (c) without and (d) with the adhesive. Scale bars represent ~2 cm. e) Bacterial concentration after incubation. Bars represent mean values, error bars denote the standard deviation as calculated from *n* = 3 biological replicates.

**Figure S20.** Lap shear strength values of birch wood samples which were repaired/reglued for a second time. The bar represents the mean value, the error bar denotes the standard deviation as calculated from *n* = 5 samples.

**Heat gun treatment of the adhesive:** As an alternative method for the curing step, a heat gun treatment (Steinel HL500s, 220V, 1500 W) was applied to the adhesive on the stainless-steel samples. First, similar amounts of adhesive were placed onto the ends of one side of two stainless steel samples. Then, the heat gun was switched on at the first power setting (~300 °C, 240 L min^–1^) and pre-heated for 2 min. After that, the nozzle of the heat gun was placed vertically (at a distance of 1 cm) above the end of the steel sample carrying no adhesive, and the steel sample was heated up for 40 s (thus indirectly heating the adhesive). Afterwards, the two steel samples were slightly moved against each other to distribute the adhesive uniformly across the interface and then manually pressed together for ~1 min.

**Figure S21.** Lap shear strength of bonded stainless steel samples, where the adhesive was cured by treating it with a heat gun. The bar represents the mean value, the error bar denotes the standard deviation as calculated from *n* = 6 samples.

**Figure S22.** Lap shear experiments conducted on birch wood samples using different ratios of the supporting glue components. CA indicates a citric acid substitution for malic acid (MA). ‘TA’ denotes tannic acid. All formulations shown exhibit lower average lap shear strength values than the glue composition of 80MA80TA used in this study. Bars represent mean values, error bars denote the standard deviation as calculated from *n* = 8 samples.

**Figure S23.** Photograph of a custom-made sample holder mounted into the Instron device. This bespoke sample holder was designed to prevent any bending of samples during the compression tests to ensure accurate measurements.
